# The Function of a Fever Hospital

**Published:** 1895-05-25

**Authors:** 


### The Institutional Workshop.

### THE FUNCTION OF A FEVER HOSPITAL.

(From a Medical Correspondent.)

In the natural history of epidemics there are few points more curious than the way in which an outbreak, which has spread through a population despite all that could be done to limit its progress, in the end takes a turn, and dies away as rapidly as it had commenced. If the spread of an epidemic depended entirely upon the multiplicity of the opportunities of infection this clearly would not be the case. Yet it is largely on the assumption that this is so, and that epidemics can be controlled by isolation, that sanitary authorities all over the country are urging the erection of hospitals for the treatment of infectious diseases, and in fact this assumption has so far become a part of our sanitary faith that the Act under which the money for this purpose is being spent is called the Isolation Hospitals Act.

Clearly then it is a matter of very considerable importance that we should quickly arrive at some definite conclusion as to the real purposes of hospitals for infectious diseases, for on that to some extent depends their size.

Put shortly, then, the suggestion is, that the dying down of an epidemic, say, of scarlet fever, is due to seasonal influences and to the lessening of the mass of susceptible individuals, rather than to any diminution in the number of starting points of infection consequent on the cases being treated in hospital. At the very time when the prevalence of 1893 began to subside there were far more cases outside the hospital walls, more *foci* of infection, than in the early part of the year when it began to rise. The opportunities for infection were greater than ever, the hospital system had completely broken down as a means of isolation, and yet, in deference to the regular seasonal habit of the disease, the excessive prevalence died away. It certainly was not the hospital isolation that killed the epidemic.

Among the various causes which conduce to the cessation of an epidemic of infectious disease one of the most potent is the diminution of the susceptibility of the population, owing to the number of persons rendered immune, at any rate for the time, by having gone through an attack. This is also a factor in the production of that varying prevalence of scarlet fever, in periods of a few years, which is commonly recognised as a feature in its natural history. When a sufficiently large population of children under five years of age has accumulated, then if infection arises it spreads; when that batch of children has become sufficiently immune, then the epidemic dies—hospital or no hospital.

This brings us round to the question, What is the proper function of our hospitals for infectious diseases, more especially in regard to scarlet fever and diphtheria? Are they to be looked on, as is done almost universally by sanitary authorities, merely as a means for isolating patients who otherwise would become *foci* of infection, and are we, therefore, to consider our sanitary machinery deficient unless it provides facilities for isolating all the cases that occur, or should we not rather regard our hospitals as places in which, on the one hand, patients may safely pass through their malady and gain the desired immunity from further attack, and in which, on the other hand, by careful treatment the type of the disease may be kept at a low degree of virulence.

The question of "type" is a most important one. A disease which at one period is apt to be an extremely mild affair may at another period attain a high degree of virulence, not only causing a high death-rate among those normally susceptible to it, but by its increased infectiveness attacking persons who otherwise would have proved immune. Now this at least we may hope, that by the removal and careful treatment of the more severe cases of scarlet fever, and especially of cases whose hygienic surroundings are bad, the disease may be prevented from assuming its more virulent form; and, after all, this is the most important matter. The Metropolitan Asylums Board, with a certain complacency, insert in their report a comparison of the London death-rate from scarlet fever both before and after the opening of their hospitals, pointing out that while during the 13 years from 1859 to 1871 the death-rate from scarlet fever per 1,000 of population was 1·07, it was only 0·29 per 1,000 in the 13 years just past. Knowing the history of past epidemics of scarlet fever, we cannot assert with confidence that the hospitals have had anything to do with this result; but we can feel fairly sure that, so far as they are to be credited with it, so important an improvement cannot be fairly attributed to isolation, for not until last year were they ever able to isolate even half the patients affected. As a matter of fact, in 1893 the Board isolated 14,548 patients suffering from scarlet fever, and between that year and 1894 the scarlet fever mortality in London dropped from 0·37 to 0·22 per 1,000. But an even greater fall took place between 1884 and 1885, viz., from 0·36 to 0·18, when less than two thousand cases were being isolated. There is evidently something else besides isolation at work, and we may fairly believe that the removal of patients from the crowded and insanitary dwellings, where virulence of type is bred, and their treatment amid good surroundings, have been far more important factors than mere isolation in producing the undoubted diminution which is observable during recent years in the mortality of scarlet fever.

The moral of this is obvious. The present sanitary theory is that every case of scarlet fever which cannot be properly isolated at home should be removed to hospital. A hopeless task! If the Asylums Board with their unlimited funds found themselves overwhelmed in 1893, when only 0·37 in every 1,000 inhabitants were dying of scarlet fever, how would they meet an epidemic like that of 1870 when the mortality was 1·88, or five times as heavy? How are other sanitary authorities with their limited means to undertake so onerous a duty?

A modification of the type of disease is not, however, so hopeless a task. Virulence is bred amid squalor, want, and insanitary surroundings, whereas in a wellappointed hospital the disease loses, to a large extent, its danger and even its infectiveness; and we can quite agree with the *British Medical Journal* in the remark that, "although the stamping out of scarlet fever by universal isolation is a dream of some, a lessening of the virulence of type is probably much more within the reach of practical medicine."

For this purpose the hospitals should be properly used. Under the isolation hypothesis the tendency has been to open them to every one, and it is to be feared that not uncommonly cases of exceeding mildness have been taken to the exclusion of others, which, from their severity and more especially from their surroundings, were likely to become *foci* of a more virulent type. This should not be. Immediate removal is a matter in regard to which the Ambulance Committee justly take to themselves much pride, but as a means of checking an epidemic judicious selection of cases by the medical officer of health is of far more importance.

Treatment rather than isolation should be the first function of the hospital, and the careful selection of cases for removal should be regarded as a duty by the sanitary authority.

